# Comparison of Elixhauser and Charlson Methods for Predicting Oral Cancer Survival

**DOI:** 10.1097/MD.0000000000002861

**Published:** 2016-02-18

**Authors:** Heng-Jui Chang, Po-Chun Chen, Ching-Chieh Yang, Yu-Chieh Su, Ching-Chih Lee

**Affiliations:** From the Department of Radiation Oncology, Min-Sheng General Hospital, Taoyuan (H-JC); Department of Radiation Oncology, Pingtung Christian Hospital, Pingtung (P-CC); Department of Radiation Oncology, Chi-Mei Medical Center, Tainan (C-CY); Department of Hema-Oncology, Dalin Tzu Chi Hospital, Buddhist Tzu Chi Medical Foundation, Chiayi (Y-CS); Department of Otorhinolaryngology, Head and Neck Surgery, Kaohsiung Veterans General Hospital, Kaohsiung (C-CL); Department of Otolaryngology, Head and Neck Surgery, Tri-Service General Hospital (C-CL); and School of Medicine, National Defense Medical Center (C-CL), Taipei, Taiwan.

## Abstract

Cancer survival correlates not only with the features of primary malignancy but also with the degree of underlying comorbidities. Of the multiple methods used for evaluating the impact of comorbidities on survival, the Charlson and Elixhauser methods are most common. This study compared these 2 comorbidity measures for predicting survival in oral cancer patients.

Using the Taiwan National Health Insurance claims data (2008–2011), we acquired data regarding patients’ characteristics, comorbidities, and survival from 3583 oral cancer patients. Comorbidity was classified according to both the Charlson comorbidity and Elixhauser comorbidity based on the International Classification of Diseases, 9th Revision. The Elixhauser comorbidity score and Charlson comorbidity score were also calculated. The prediction of survival was determined using measures of discrimination, including the Akaike information criterion and Harrell C (C-statistic).

The mean age of the study cohort was 52 ± 10 years, and 94.9% of the patients were male. The median follow-up time was 30.1 months, and the 3-year overall survival was 61.6%. Elixhauser comorbidity method added higher discrimination, compared with the Charlson comorbidity method (Harrell C, 0.677 vs 0.651). Furthermore, the Elixhauser comorbidity score outperformed the Charlson comorbidity score in continuous variable (Harrell C, 0.654 vs 0.646) and category (Harrell C, 0.658 vs 0.645).

The Elixhauser method is a superior comorbidity risk-adjustment model for oral cancer survival prediction. Utilization of the Elixhauser comorbidity method may be encouraged for risk adjustment in oral cancer study.

## Abstract 2

Supplemental Digital Content is available in the text

## INTRODUCTION

Mary Charlson and other authors defined 19 clinical diagnoses in a narrow population of fewer than 700 patients in 1987 by reviewing hospital charts and assessing their relevance to 1-year mortality. Each diagnosis was assigned a weighting score, and the index was the sum of all scores. This index has been validated by several studies^1–3^ and is the most widely used comorbidity assessment used over the last several decades. Multiple adaptations of the Charlson method have been created, including modifications to 17 categories (Charlson/Deyo),^4^ modification of a specific International Classification of Diseases, Clinical Modifications (ICD-CM) diagnosis codes (Charlson/Romano),^5^ and a translation from ICD-9 codes to ICD-10 codes.^6,7^

Another popular assessment method was introduced by Elixhauser et al in 1998^8^ based on 1,779,167 California inpatient datasets. A more comprehensive set of 30 comorbidity measures was developed. The Elixhauser comorbidity method is able to predict in-hospital mortality, length of hospitalization, and hospital charges. A comorbidity with pre-existing diagnosis before admission should be distinguished from a complication acquired during the hospital stay or later treatment.

Growing evidence has found the Elixhauser method to be superior to the Charlson method for risk-adjustment. Lieffers et al compared the 2 methods for predicting colorectal cancer survival in a research cohort of 574 patients. They concluded that adding Charlson method to the base model did not change the discriminative power, while addition of the Elixhauser method yielded higher discrimination and C-statistics.^9^ Quail et al studied 5 comorbidity measures in 3 population-based cohorts: a general population in a province (n = 662,423), a group of patients with diabetes (n = 41,925), and a group of patients with osteoporosis (n = 28,068). The Elixhauser method resulted in the highest C-statistic, followed by the Charlson method.^10^

Oral cancer ranks the fourth most common cancer in Taiwanese males. In light of the differences between Charlson and Elixhauser assessments, we compared the Charlson and Elixhauser for determining oral cancer survival using a population-based cohort from the National Health Insurance Research Database (NHIRD). Building on a base model that included gender, radiotherapy, socioeconomic status (SES), geographic region, and urbanization, we added the Charlson and Elixhauser methods and compared the results. Our hypothesis was that the Elixhauser method would be substantially better than the Charlson method for predicting oral cancer overall survival.

## METHODS

### Ethics Statement

Ethical approval was obtained from the Institutional Review Board of Dalin Tzu Chi Hospital in Taiwan. All personal identification was encrypted in NHIRD, so the requirement for informed consent was waived.

### Data Sources and Study Populations

All data, including head and neck cancer (briefed as oral cancer) and numerous comorbidity conditions, were collected from NHIRD. Taiwan had a universal single-payer NHI program since 1995. As of 2008, 98% of Taiwan's population was enrolled in this program. All contracted providers must regularly submit claim information to get reimbursement. Large computerized databases derived and managed by the Bureau of NHI are provided to scientists in Taiwan for research purposes.^11^

The cohort included patients with cancers classified as codes 140 to 145 according to the International Classification of Diseases, 9th Revision, Clinical Modifications (ICD-9-CM) for oral cancer except that codes 142 (malignant neoplasm of major salivary glands) was excluded. Patients eligible for the study were those with stage I to IVB oral cancer proven by biopsy or surgery who underwent wide resection of tumor and free flap reconstruction. A total of 3583 patients seen from January 2008 to December 2011 were selected.

Diagnoses of comorbid diseases defined in the Charlson and Elixhauser methods were also identified, and the related ICD-9-CM codes are shown in Supplementary Tables 1 and 2. There were some coding differences in the similar diagnosis between the 2 methods. For example, rheumatic disease (ICD-9-CM: 710.0, 710.1, 710.4, 714.0–714.2, 714.81, 725.x) defined by the Charlson method differed slightly from rheumatoid arthritis/collagen vascular diseases (ICD-9-CM: 701.0, 710.x, 714.x, 720.x, 725.x) defined by the Elixhauser method.

Patient characteristics included gender, mean age, receiving chemotherapy, receiving radiotherapy, SES (low, moderate, and high), geographic region (northern, central, southern, and eastern), community type (urban, suburban, and rural), and the teaching level of the medical facility (medical center, regional, and district). Evaluation of the SES was based on incomes in Taiwan and several urbanization variables.^12^

### Comorbidity Methods

We compared the 2 comorbidity methods, Charlson and Elixhauser, and identified the comorbid conditions included by each. The Charlson method included 17 categories, while the Elixhauser method included 30. We further calculated the weighted Charlson comorbidity score and Elixhauser comorbidity score as previous literature mentioned.^9,13^

### Statistical Analysis

The entry time of this cohort started on January 1, 2008 and the end-of-study date was December 31, 2011. Three-year overall survival was calculated in this cohort. Death from cancer was recorded as an event, and subjects were censored if they lived longer than 3 years.

The Akaike information criterion (AIC) and Harrell C (C-statistic) were used to assess predictive performance and evaluate discrimination against base model parameters (gender, radiotherapy, SES, geographic regions, teaching level of hospital, and urbanization). The AIC was calculated as AIC = 2k − 2 ln(L), where k is the estimated parameter in the model and L is the maximum likelihood. The smaller the AIC, the better the predictive ability of the model. The Harrell C-statistic measures how well the model can discriminate between observations, with possible values of 0.5 (no predictive ability), 0.7 to 0.8 (acceptable), 0.8 to 0.9 (excellent), 0.9 to 1.0 (outstanding), 1 (perfect discrimination).^14^*P* < 0.05 was considered significant. All analyses were performed using SPSS (version 15, SPSS, Inc., Chicago, IL) statistical packages. Variables or data of the 2 comorbidity methods can be represented as continuous scale, or in categorical or discrete forms. We also used the ways of discrete variable (item), continuous variable, and category to evaluate the AIC and Harrell C-statistic of each analytic group. The empirical quartiles method and concept was applied to investigate the categorized scores. For example, score 0 was set as 1 quantile, score 4 was another quantile, and so on. We could create different category groups based on these quantiles.

## RESULTS

We studied 3583 oral cancer patients, with a median follow-up duration of 30.1 months, and the 3-year overall survival was 61.6%. Median survival was not reached at the end of study. The cohort comprised 5.1% females and 94.9% males. The mean age of these patients was 52 ± 10 years. Patients in the cohort had been treated using chemotherapy (33.1%) and/or radiotherapy (5.4%). The cohort included subjects of low (42.1%), moderate (38.2%), and high (19.7%) SES who lived in urban (20.7%), suburban (44.1%), and rural (35.2%) settings. The majority of the patients (75.2%) were from medical centers, while 23.5% were diagnosed in regional hospitals (Table 1).

**TABLE 1:**
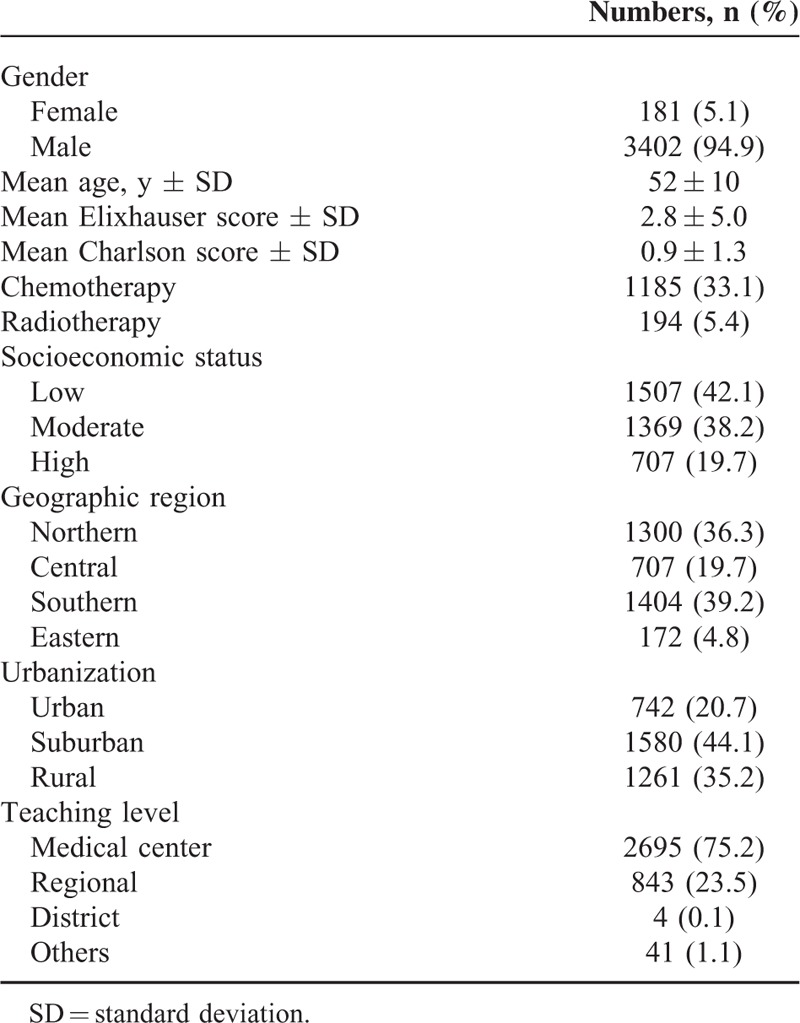
Demographic and Clinical Characteristics of Study Patients (n = 3583)

The distribution of comorbidities based on the 2 comorbidity methods is shown in Tables 2 and 3. Of the Elixhauser comorbidities, hypertension (26.3%) was the most common item, followed by uncomplicated diabetes (18.6%), solid tumor without metastasis (13.6%), and deficiency anemia (11.3%). In contrast, the Charlson comorbidities ranked the 1st to 3rd most common items as diabetes mellitus without end-organ damage (18.7%), any malignancy including lymphoma and leukemia (13.8%), and peptic ulcer disease (11.7%).

**TABLE 2:**
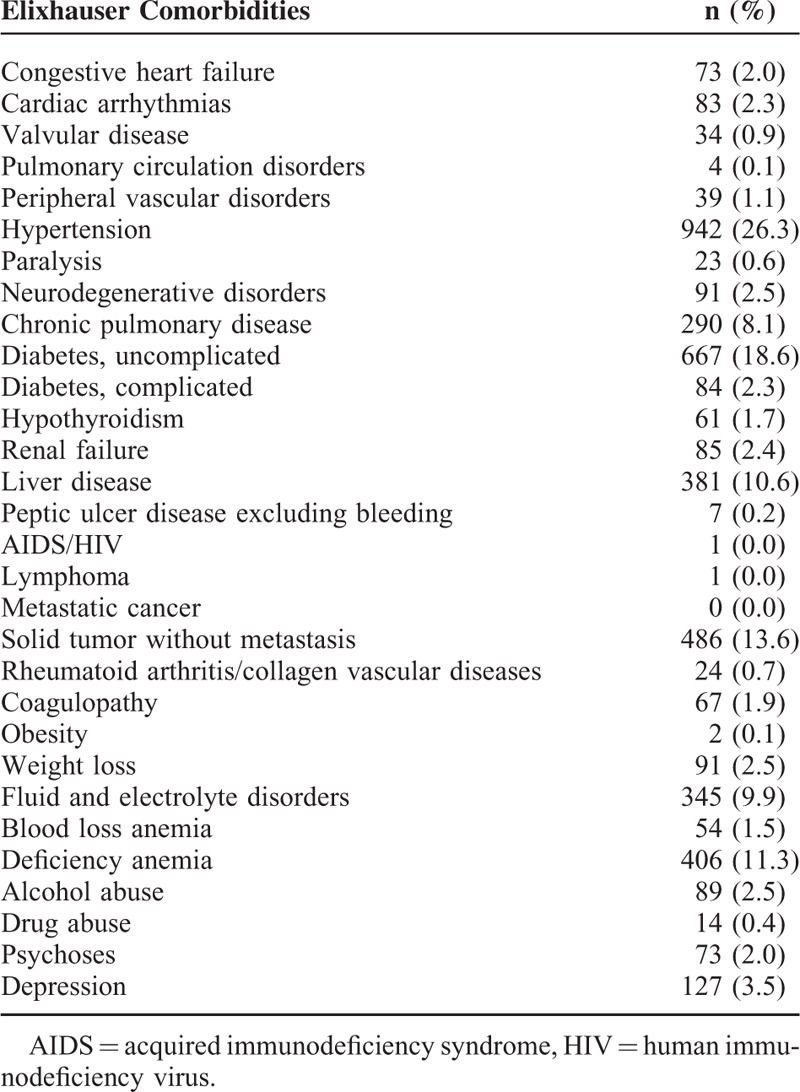
Distribution of Elixhauser Comorbidities in Patient Cohort (n = 3583)

**TABLE 3:**
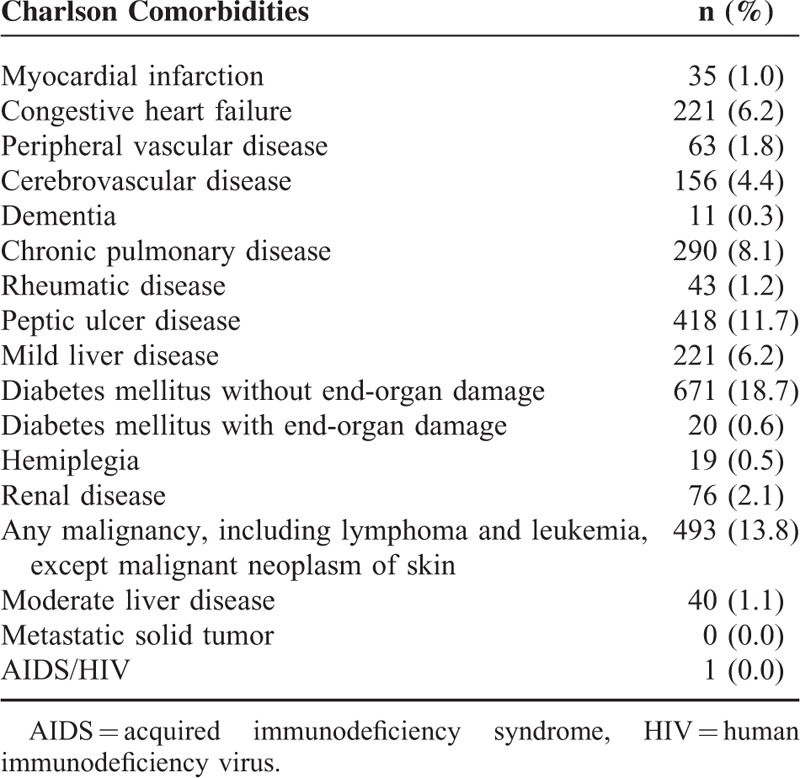
Distribution of Charlson Comorbidities in Patient Cohort (n = 3583)

In multivariable (MV) adjusted hazard ratio (aHR) analysis of Elixhauser conditions, congestive heart failure (aHR, 1.78; 95% confidence interval [CI]: 1.26–2.52), neurodegenerative disorders (aHR, 1.74; 95% CI: 1.34–2.25), solid tumor without metastasis (aHR, 1.24; 95% CI: 1.08–1.44), obesity (aHR, 4.97; 95% CI: 1.16–21.29), weight loss (aHR, 1.44; 95% CI: 1.18–1.88), fluid and electrolyte disorders (aHR, 1.96; 95% CI: 1.69–2.28), deficiency anemia (aHR, 1.37, 95% CI: 1.18–1.60), and depression (aHR, 1.30; 95% CI: 1.01–1.68) were associated with increased mortality risk after adjusting variables. We also found 3 protecting factors that had significantly lower risk of death: hypertension (aHR, 0.85; 95% CI: 0.74–0.97), hyperthyroidism (aHR, 0.59; 95% CI: 0.37–0.93), and rheumatoid arthritis/collagen vascular diseases (aHR, 0.18; 95% CI: 0.04–0.75; Table 4).

**TABLE 4:**
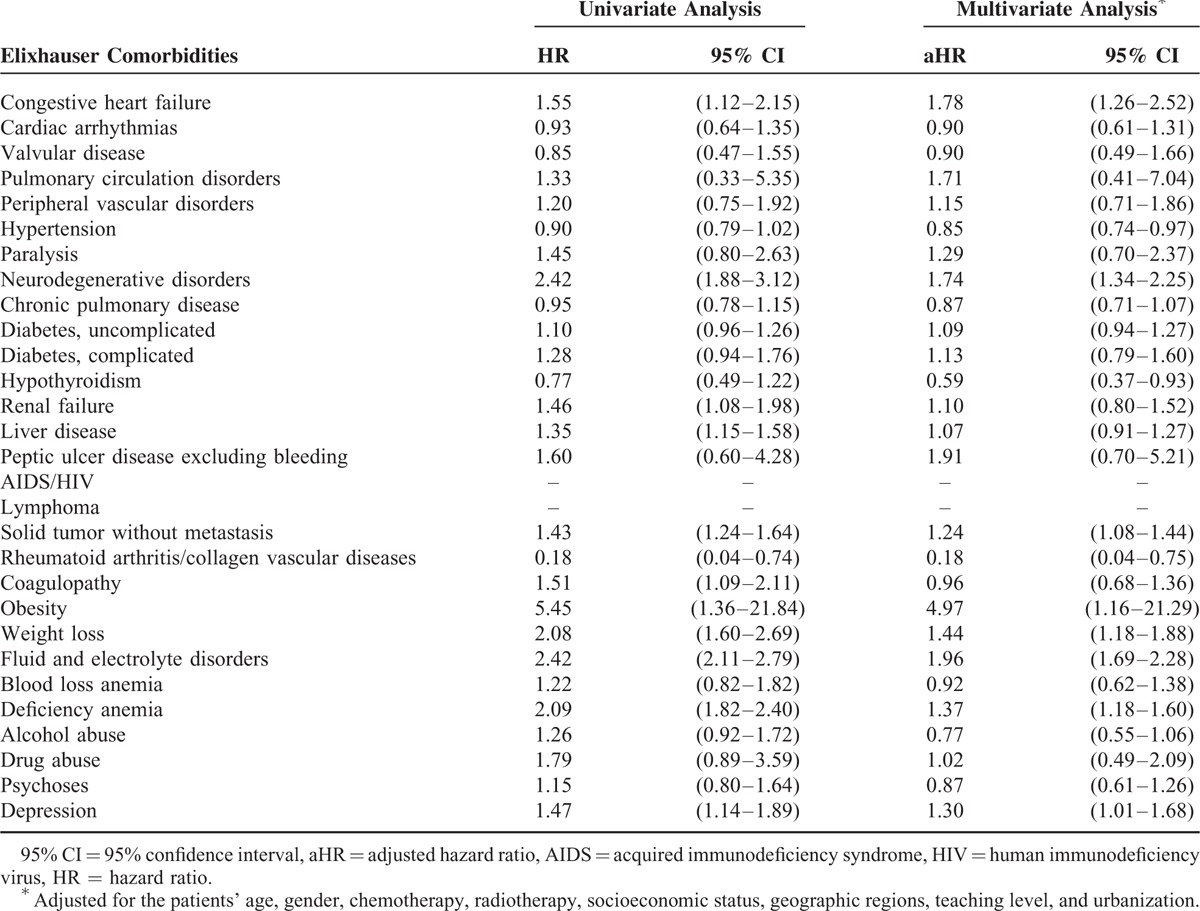
Adjusted Hazard Ratios of Mortality Among Oral Cancer Patients (2008–2011)

In MV aHR evaluation of Charlson conditions, several risk factors were distinguished, including congestive heart failure (aHR, 1.49; 95% CI: 1.22–1.82), any malignancy except skin neoplasm (aHR, 1.16; 95% CI: 1.01–1.34), and moderate liver disease (aHR, 1.65; 95% CI: 1.07–2.55). We did not identify any protecting factor in Charlson conditions (Table 5). We also used Elixhauser and Charlson comorbidity scores as prediction methods. Supplementary Tables 3 and 4, show the results.

**TABLE 5:**
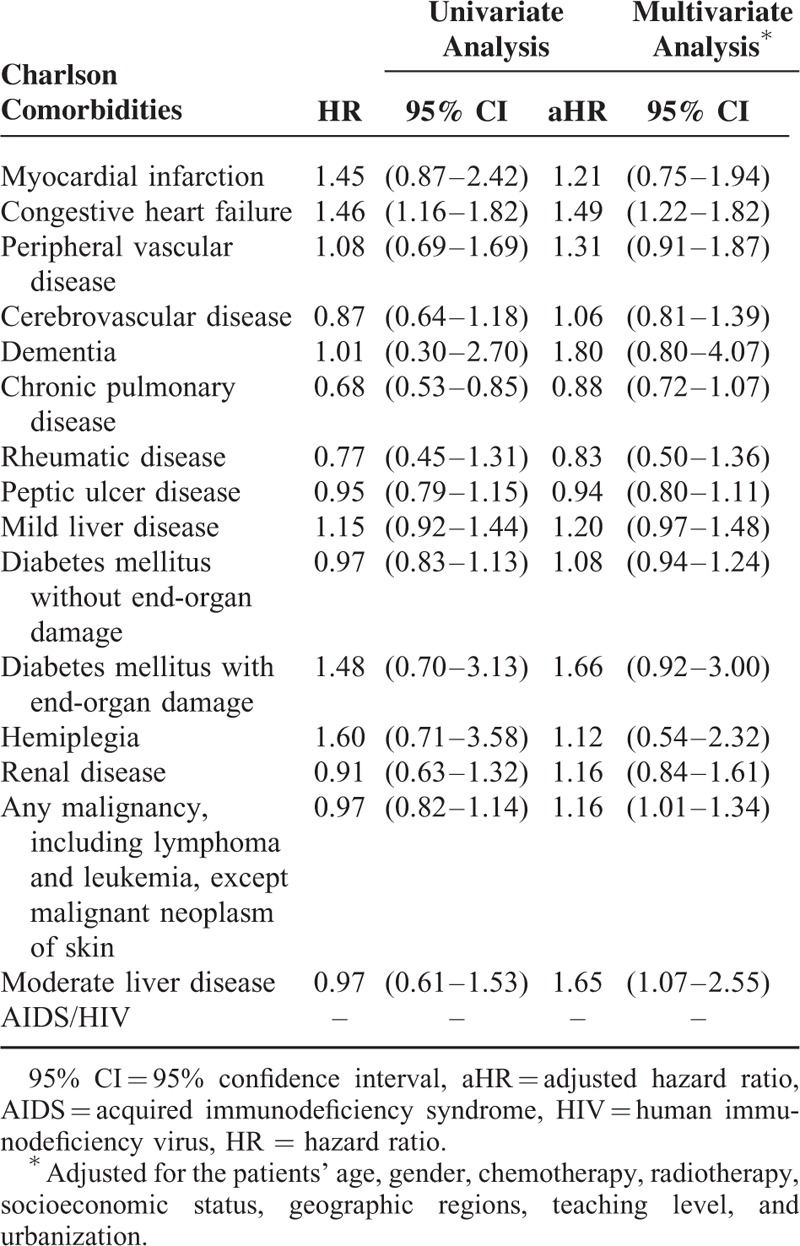
Adjusted Hazard Ratios of Mortality Among Oral Cancer Patients (2008–2011)

Table 6 shows the Harrell C-statistics for the Elixhauser and Charlson comorbidity methods adjusted for age, gender, adjuvant therapy, SES, geographic region, urbanization of residence, and hospital's teaching level for 3-year survival. All patients (n = 3583) are separated into 2 groups: surgery alone (n = 2377) and surgery and adjuvant therapy (n = 1206). No matter using item, continuous variable, or category for analysis, Elixhauser method is comprehensively a better comorbidity risk adjustment with higher Harrell C-statistic and lower AIC. For example, Elixhauser comorbidity method in item analysis added higher discrimination, compared with the Charlson comorbidity method (Harrell C, 0.677 vs 0.651). Furthermore, the Elixhauser comorbidity score outperformed the Charlson comorbidity score in continuous variable (Harrell C, 0.654 vs 0.646) and category (Harrell C, 0.658 vs 0.645). When summation of the comorbidities as a weighted single score, Elixhauser method performed better than Charlson method.

**TABLE 6:**
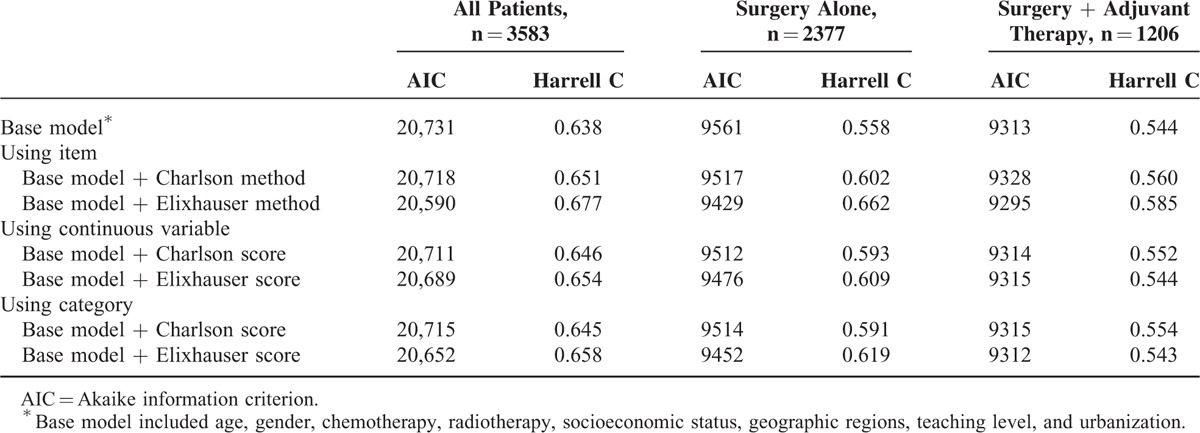
Comparison of Charlson and Elixhauser Comorbidities With Respect to 3-y Survival

## DISCUSSION

In this population cohort study, we found that the Elixhauser method was a superior comorbidity risk-adjustment method than Charlson method, with a significantly higher Harrell C statistics for oral squamous cell carcinoma patients. A study from Austin et al depicted that researchers can use comorbidities individually or through the summary measures when adjusting for comorbidities in statistical models. Their study further validated and confirmed the utility of the summary comorbidity measures as substitutes for use of the individual comorbidity variables.^15^ In our study, we used both the individual variables and the summation scores for analysis, ant the result showed that Elixhauser method performed better than Charlson method in both ways. The strengths of this study included its population-based database that included both outpatient and inpatient data. Our oral cancer patients were composed of all ages, through different stages (I–IVB), with all-cause mortality. Using Harrell C statistics, our observation that Elixhauser method outperformed Charlson method in predicting survival was consistent with the results of other studies. When using a summation score, the Elixhauser comorbidity index score was also a superior risk-adjustment model compared with the Charlson comorbidity index score.

Comorbid illnesses can affect the outcome of cancer in multiple ways, including altering the clinical course of cancer and affecting the choice of treatment. Chronic comorbidities usually involve gradual processes that take time to manifest their long-term effects. In advanced stage cancer or disseminated disease in a rapid progression, these chronic effects might not be seen. Our study results confirm those of Read et al, who observed that comorbidity was prognostically of the greatest significance among cancers with the highest survival and least important in those with the worst survival.^16^ Another population-based cohort study from Reid et al similarly concluded that the magnitude of the comorbidity effect was lower in advanced stage head and neck cancer.^17^ In a head and neck cancer study from Alho et al,^18^ the underlying probability of death was lower in early stage and young age patients, so the comorbidities were prognostically more important.

We found that 3 comorbidities were independently associated with lower mortality risk. Patients who had uncomplicated hypertension, hyperthyroidism, rheumatoid arthritis, or collagen vascular diseases were likely to be healthier, and had better survival. Similar protective factors were also observed by Elixhauser et al^8^ and by Johnston et al^19^ in a study of intensive care patients. These studies indicated that hypertension, diabetes mellitus, depression, anemia, and cardiac valvular disease were associated with a decreased risk of death. Elixhauser et al explained that sometimes patients with catastrophic illness have so many diagnoses that nonthreatening diagnoses are not coded. Inversely, a healthy patient with a low risk of death is more likely to have such diagnoses in the absence of more serious diseases. Consequently, the presence of codes for nonthreatening diseases is indicative of a relatively healthy patient.

Our study has several limitations. First, the Elixhauser classification system requires 30 binary variables, making its use for reporting and analyzing comorbidities cumbersome. Thus, these results may not be generalizable to other population groups or outcome measures. Second, the accuracy of assigning diagnostic codes might be variable, leading to coding bias. Possible sources of inconsistency include the accuracy of code design, physician documentation, and financial pressures that could influence the capture of comorbidities based on how they are remunerated. Data indicate that comorbid conditions might be under-assigned in claims as compared to those assigned in medical records.^20^

## CONCLUSIONS

The Elixhauser comorbidity method is an adequately discriminative comorbidity index for risk adjustment and is superior to the Charlson method in predicting survival of oral cancer patients. The Elixhauser comorbidity method outperformed the Charlson method in both the single comorbidity adjustment way and a weighted score method.

## Supplementary Material

Supplemental Digital Content
