# Supplementary material for: Comparison of Elixhauser and Charlson Methods for Predicting Oral Cancer Survival
Source: Medicine (Baltimore). 2016 Feb 18;95(7):e2861. doi: 10.1097/MD.0000000000002861 (PMC4998653; doi:10.1097/MD.0000000000002861)
Supplement: Supplemental Digital Content [file medi-95-e2861-s001.pdf]

Supplementary Table 1. Comorbidity distribution based on the Elixhauser comorbidities

Comorbidity	Elixhauser's ICD-9-CM
Congestive heart failure	398.91, 402.01, 402.11, 402.91, 404.01, 404.03, 404.11, 404.13, 404.91, 404.93, 428.x
Cardiac arrhythmias	426.10, 426.11, 426.13, 426.2-426.53, 426.6-426.8, 427.0, 427.2, 427.31, 427.60, 427.9, 785.0, V45.0, V53.3
Valvular disease	093.2, 394.x-397.1, 397.9, 424.x, 746.3-746.6, V42.2, V43.3
Pulmonary circulation disorders	416.x, 417.9
Peripheral vascular disorders	440.x, 441.x, 442.x, 443.1-443.9, 447.1, 557.1, 557.9, V43.4
Hypertension	401.1, 401.9, 642.0,401.0, 402.x-405.x, 642.1, 642.2, 642.7, 642.9
Paralysis	342.x-344.x, 438.2-438.5
Other neurological disorders	330.x-331.x. 332.0, 333.4, 333.5, 334.x, 335.x, 340, 341.1-341.9, 345.x, 347.x, 780.3, 784.3
Chronic pulmonary disease	490x-492.x, 493.x, 494x-505.x, 506.4
Diabetes, uncomplicated	250.0-250.3, 648.0
Diabetes, complicated	250.4-250.9, 775.1
Hypothyroidism	243-244.2, 244.8, 244.9
Renal failure	403.01, 403.11, 403.91, 404.02, 404.03, 404.12, 404.13, 404.92, 404.93, 585.x, 586.x, V42.0, V45.1, V56.x
Liver disease	070.22, 070.23, 070.32, 070.33, 070.44, 070.54, 456.0, 456.1, 456.20, 571.0, 571.2-571.9, 572.3, 572.8, V42.7
Peptic ulcer disease excluding bleeding	531.41, 531.51, 531.61,531.7, 531.91, 532.41,532.51, 532.61, 532.7,532.91, 533.41, 533.51,533.61, 533.7, 533.91,534.41, 534.51, 534.61,534.7, 534.91
AIDS/H1V	042.x-044.x
Lymphoma	200.x-202.3, 202.5-203.0,203.8, 238.6, 273.3
Metastatic cancer	196.x-199.x
Solid tumor without metastasis	140.x-172.x, 174.x, 175.x,179.x-195.x
Rheumatoid arthritis/ collagen vascular diseases	701.0, 710.x, 714.x, 720.x,725.x
Coagulopathy	286.x, 287.1, 287.3-287.5
Obesity	278.0
Weight loss	260.x-263.x, 783.2
Fluid and electrolyte disorders	276.x
Blood loss anemia	280.0, 648.2
Deficiency anemia	280.1-281.9, 285.2, 285.9
Alcohol abuse	291.0-291.3, 291.5, 291.8,291.9, 303.x, 305.0
Drug abuse	292.0, 292.82-292.89,292.9, 304.x,305.2-305.9, 648.3
Psychoses	295.x-298.x, 299.1
Depression	300.4, 301.12, 309.0, 309.1,311

Supplementary Table 2. Comorbidity distribution based on the Charlson comorbidities

Comorbidity	ICD-9-CM
Myocardial infarction	410.x, 412.x
Congestive heart failure	428.x
Peripheral vascular disease	443.9, 441.x, 785.4, V43.4
Cerebrovascular disease	430.x-438.x
Dementia	290.x
Chronic pulmonary disease	490.x-505.x, 506.4
Rheumatic disease	710.0, 710.1, 710.4,714.0-714.2, 714.81, 725.x
Peptic ulcer disease	531.x-534.x
Mild liver disease	571.2, 571.4-571.6
Diabetes mellitus without end-organ damage	250.0-250.3, 250.7
DM with end-organ damage	250.4-250.6
Hemiplegia	344.1, 342.x
Renal disease	582.x, 583-583.7, 585.x, 586.x,588.x
Any malignancy, including lymphoma and leukemia, except malignant neoplasm of skin	140.x-172.x, 174.x-195.8,200.x-208.x
Moderate liver disease	456.0-456.21, 572.2-572.8
Metastatic solid tumor	196.x-199.1
HIV/AIDS	042.x-044.x

Supplementary Table 3. The adjusted hazard ratios for different comorbidity scores (using continuous variable)

	Elixhauser score model		Charlson score model	
	Adjusted HR	95% CI	Adjusted HR	95% CI
Comorbid condition	1.03	(1.02-1.04)	1.09	(1.05-1.14)
Age	1.00	(0.99-1.01)	1.00	(0.99-1.01)
Gender				
Male	1		1	
Female	0.80	(0.61-1.05)	0.80	(0.61-1.06)
Chemotherapy				
No	1		1	
Yes	2.72	(2.43-3.05)	2.75	(2.46-3.09)
Radiotherapy				
No	1		1	
Yes	0.88	(0.71-1.10)	0.91	(0.74-1.13)
Socioeconomic status				
Low / Moderate SES	1		1	
High SES	0.85	(0.74-0.98)	0.84	(0.72-0.97)
Geographic Region				
Northern/ Central	1		1	
Southern/ Eastern	0.95	(0.84-1.07)	0.96	(0.85-1.09)
Urbanization				
Urban/ Suburban	1		1	
Rural	0.94	(0.83-1.06)	0.94	(0.83-1.06)
Teaching level				
Medical center	1		1	
Others	0.97	(0.85-1.10)	0.99	(0.97-1.13)

Abbreviations: HR, hazard ratio; CI, confidence interval

Supplementary Table 4. The adjusted hazard ratios for different comorbidity scores (using category)

	Elixhauser score model		Charlson score model	
	Adjusted HR	95% CI	Adjusted HR	95% CI
Comorbid condition				
Low	1		1	
Moderate	1.30	(1.11-1.52)	1.16	(1.00-1.33)
High	1.78	(1.57-2.01)	1.33	(1.18-1.52)
Age	1.00	(0.99-1.00)	1.00	(0.99-1.01)
Gender				
Male	1		1	
Female	0.81	(0.62-1.07)	0.80	(0.61-1.05)
Chemotherapy				
No	1		1	
Yes	2.63	(2.34-2.95)	2.74	(2.44-3.07)
Radiotherapy				
No	1		1	
Yes	0.89	(0.72-1.11)	0.91	(0.74-1.13)
Socioeconomic status				
Low / Moderate SES	1		1	
High SES	0.85	(0.74-0.99)	0.83	(0.72-0.96)
Geographic Region				
Northern/ Central	1		1	
Southern/ Eastern	0.96	(0.84-1.08)	0.96	(0.85-1.09)
Urbanization				
Urban/ Suburban	1		1	
Rural	0.93	(0.83-1.06)	0.95	(0.84-1.07)
Teaching level				
Medical center	1		1	
Others	0.96	(0.84-1.09)	0.99	(0.87-1.13)

Abbreviations: HR, hazard ratio; CI, confidence interval
